# Muscling in on the third dimension

**DOI:** 10.7554/eLife.06430

**Published:** 2015-02-16

**Authors:** Mohsen Afshar Bakooshli, Penney M Gilbert

**Affiliations:** Institute of Biomaterials and Biomedical Engineering, University of Toronto, Toronto, Canada; Institute of Biomaterials and Biomedical Engineering, University of Toronto, Toronto, CanadaPenney.Gilbert@utoronto.ca

**Keywords:** tissue engineering, human skeletal muscle, contractile force, muscle physiology, drug testing, human

## Abstract

The development of a functional three-dimensional model of human skeletal muscle tissue could accelerate progress towards new and personalized treatments for skeletal muscle disorders.

**Related research article** Madden L, Juhas M, Kraus WE, Truskey GA, Bursac N. 2015. Bioengineered human myobundles mimic clinical responses of skeletal muscle to drugs. *eLife* **4**:e04885. doi: 10.7554/eLife.04885

**Image** The cells in muscle tissue are aligned with one another and embedded within a dense web of proteins that acts as a scaffold

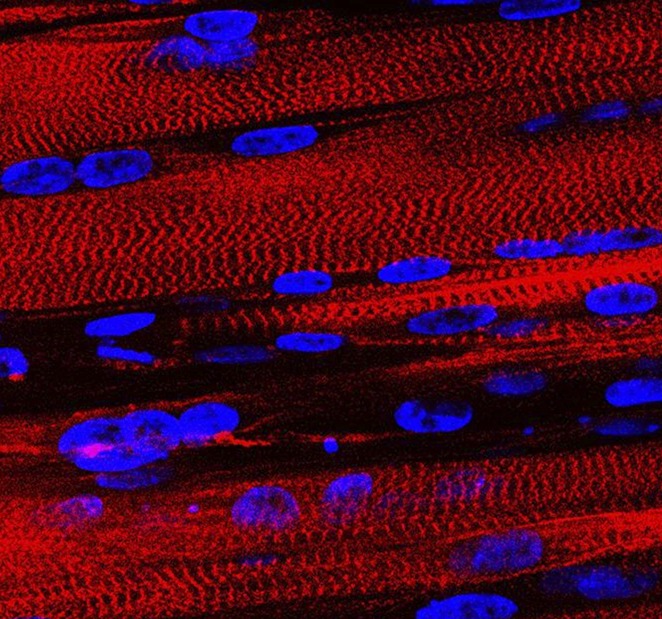

When building a house, ‘framing’ involves fitting together wooden or steel supports to give the house shape and to establish the dimensions of the space within it. The extracellular matrix—a dense web of proteins—serves a similar scaffolding role for tissues in the body. And having height, width and depth also really matters when it comes to tissues.

In the laboratory it is common to grow cells in a single two-dimensional layer on a plastic culture dish. This strategy has expanded our understanding of cells, but has often failed to lead to new therapies to treat the diseases and disorders that can afflict our tissues. In contrast, three-dimensional culture models of human skin, lung, cardiac tissue and liver faithfully mimic the responses of living tissue, and can be used to predict how these tissues might respond to potential therapies (Bhatia and Ingber, 2014). This is because being three-dimensional provides tissue with stability, acts as a scaffold for repair, and even seems to be involved in the progression of certain diseases (Pampaloni et al., 2007; Lancaster and Knoblich, 2014). It is not a surprise that there is a push within the tissue-engineering community to establish three-dimensional culture models of each and every human tissue.

Skeletal muscle is the most abundant tissue in the human body and is needed for moving limbs, blinking, swallowing, breathing and maintaining a constant body temperature. It is composed of many muscle cells that are aligned with one another and embedded within an extracellular matrix scaffold. Human muscle cells form as a result of many single cells fusing with one another to create a single long cylinder; this process was first recreated in two-dimensional culture dishes in the early 1980s (Blau and Webster, 1981). However, skeletal muscle contracts in response to electrical signals from the brain, and it has been notoriously difficult to grow human muscle fibers that have this property.

Now, in *eLife,* Nenad Bursac of Duke University and colleagues—including Lauran Madden as first author—report the first three-dimensional culture model of human skeletal muscle that responds to electrical and biochemical stimulation just like the real thing (Madden et al., 2015). First, Bursac, Madden and co-workers placed human muscle cells (which had been collected from biopsies of patients) in a two-dimensional culture dish and allowed them to undergo many rounds of cell division (Figure 1). They then mixed the cells into a protein-rich scaffold and transferred the mixture into a custom-made silicone rubber mold where it formed a soft porous gel (similar to jello [US] or jelly [UK]). A nylon frame included in the mold provided two attachment points for the gel, mimicking the sites where skeletal muscle attaches to bone via tendons. Within just two weeks, the cells had fused together to form long aligned muscle fibers. What's more, these muscle fibers could twitch and contract!

**Figure 1.**
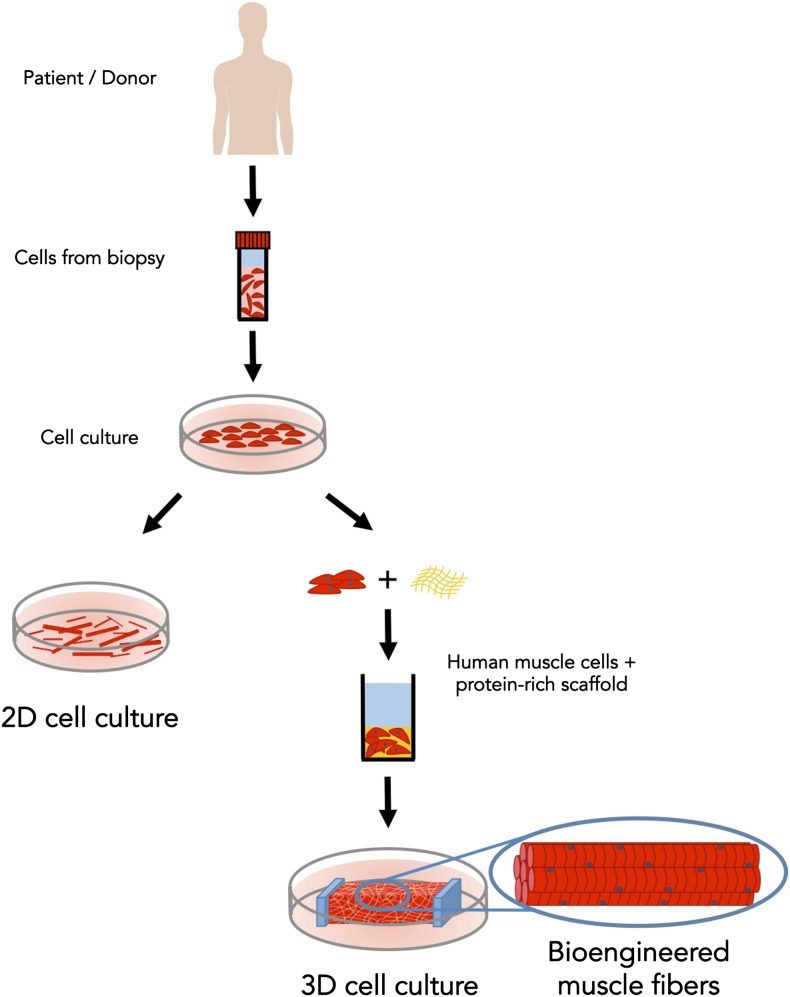
Bioengineered skeletal muscle that responds like human muscle tissue. In the early 1980s scientists figured out how to grow muscle fibers from single cells (shown in red) in a two-dimensional plastic culture dish. However, it was necessary to expose the 2D cell culture (left) to a complex mixture of molecules to make it responsive to electrical stimulation. Madden et al. have now overcome this long-standing challenge; by using a custom three-dimensional culture device (right) and with just the right protein-rich scaffold (in yellow), they generated muscle fibers that respond to electrical and biochemical cues just like normal skeletal muscle tissue.

Prior attempts by other researchers using similar devices to mimic tendons had failed to contract in response to electrical stimulation (Powell et al., 2002; Chiron et al., 2012). The secret to success seems to be in the list of ingredients used to make the protein-rich scaffold. Most laboratories had previously used collagen, a protein that is abundant in skeletal muscle. However, Madden et al. used fibrin—a product of blood clotting found in skeletal muscle undergoing repair—and found that it worked better. Fibrin had previously been shown to also be the best choice for engineering three-dimensional muscle from mouse muscle cells (Hinds et al., 2011).

Madden et al. went on to show that, when stimulated electrically or chemically, the engineered skeletal muscle responds much like normal tissue: first it releases calcium ions (Ebashi and Endo, 1968) and then it starts twitching. Furthermore, three classes of pharmaceutical drugs had similar effects on the engineered muscle as they do on normal muscle tissue in clinical settings. These results validate the potential application of the engineered skeletal muscle as a preclinical platform for drug testing.

Skeletal muscle has an amazing capacity for repair due to the presence of a small population of stem cells residing within the tissue (Mauro, 1961). However, muscle mass and function can be lost as result of degenerative conditions, like aging, and genetic conditions, such as Duchenne muscular dystrophy. The availability of a three-dimensional model of human skeletal muscle provides hope for the identification of new drugs that improve muscle strength in a diverse range of clinical settings. Moreover, it opens the door to the possibility of creating muscle tissue in a dish from a patient's own cells and then using this model to identify the most effective treatment for the patient's condition: so-called skeletal muscle personalized medicine.

With every scientific advance come new challenges. It is still unclear whether the culture system developed by Madden et al. is suitable for modeling disorders such as Duchenne muscular dystrophy. Furthermore, for pharmaceutical companies to switch to a three-dimensional tissue model, the new model must first uncover important biological findings that are obscured in standard two-dimensional cultures. And in order to integrate the new culture device with high-throughput drug screening platforms, it will be necessary to make it smaller, while also establishing simple, cost-effective metrics that can rapidly assess health of the tissue. Regardless, the advance by Bursac, Madden and co-workers has pushed the field one step closer to achieving the goal of maintaining muscle strength and health throughout life.
